# Practical Miscellanies on Diseases of Children

**Published:** 1858-05

**Authors:** L. W. Mauthner

**Affiliations:** Professor of the Diseases of Children in the University of Vienna


ART. III.—*Practical Miscellanies, being cases observed at St. Anne's Hospital for Children in Vienna.* By L. W. MAUTHNER, M.D., Professor of the Diseases of Children in the University of Vienna.*

I. *Nitrate of Silver in Serous Diarrhœa.*—Catarrhal diarrhœa was almost an epidemic disease during last autumn among the children who were admitted into this Institution. With those who were suddenly weaned, it would sometimes assume the character of cholera infantum. In the graver forms of this malady, I have prescribed crystallized nitrate of silver since 1839. To new-born infants and sucklings I give $\frac{1}{4}$ grain daily, in solution, kept in a bottle of dark glass, and administered with an ivory spoon. The remedy has always succeeded in arresting the diarrhœa without any evil consequences. The colorless stools assume a light-green hue after the second or third dose, and contain remnants of the milk taken into the stomach. As soon as the light-green stools make their appearance, I discontinue the use of the remedy. If an appropriate nourishment can now be procured for the child, especially the breast of a healthy nurse, it may be considered safe.

Thousands of experiments have confirmed me in the beneficial effects of this medicine, even in the Asiatic cholera of children, in which it also arrests the diarrhœa, given, however, in larger doses. I have convinced myself that children can take two grains of nitrate of silver daily, in this terrible disease, without any injurious effects. In most cases of Asiatic cholera it has been administered in form of clysters, in a solution of three to four grains to two ounces of water, applied every two hours.

For the cure of purulent ophthalmia of new-born infants, Professor Yaeger, Sr., has applied the nitrate of silver ever since 1837. From the highly gratifying results in that disease, I was induced to avail myself of

---

* In a letter to the Senior Editor.

it in my practice. It is now nineteen years since I observed its use, and would recommend it as an efficacious therapeutic remedy, in the diseases of children. The diarrhœa which occurs during dentition, and the much prevailing summer-complaint of America, which proves so often fatal, has been frequently cured by the administration of this remedy.

II. *Use of Ass's Milk in Atrophy of the Bones.*—On the 20th of October, soon after my arrival in Vienna from a tour through the United States, I was called to a distant part of the city to a child six months of age, who, with the exception of the head and abdomen, presented nothing but four thin staves, in the shape of extremities; a high degree of marasmus, which had been treated for four months for diarrhœa, by another physician, with extractum ligni campechiensis. I advised the abandonment of everything else, and let the child have nothing but ass's fresh milk. At the expiration of a month, the child was brought to me perfectly well and robust-looking. Next to the mother's milk, I know of nothing which acts so charmingly as the milk of the ass.

III. *Spontaneous Fractures in the graver degrees of Rachites.*—Rachites is a disease of frequent occurrence among children in this region, and often appears in the first month of infant life, in the cranium, ribs, and extremities. It seems, in most instances, to be an independent disease, originating from dynamic causes. Neither can the pressure of the brain on the occiput, nor the compression of the thorax by the atmosphere, nor, least of all, can the weight of the trunk upon the lower extremities, produce the rachitic condition.

I have often observed rachitic distortion of the extremities at an age before any attempts were made to put the legs of the children in a standing position. If such a bone is sawed through, it will immediately be perceived that the locality of the rachitic bend has its medullary canal obliterated, while an areolar tissue grows in the atrophied substance of the bone, which impedes its nutrition. In such cases it has happened that by the influence of muscles and their clonic spasms, the bone seems to be broken in these places. I have observed such fractures on several extremities, where no trace of any external violence could be discovered.

